# Coenzyme Q10 Enhances Resilience of Mitochondrial-like Membranes Against Amyloidogenic Peptides

**DOI:** 10.3390/membranes15050148

**Published:** 2025-05-13

**Authors:** Raina Marie Seychell, Adam El Saghir, Gianluca Farrugia, Neville Vassallo

**Affiliations:** 1 Department of Physiology and Biochemistry, Faculty of Medicine and Surgery, University of Malta, MSD 2080 Msida, Malta; 2 Centre for Molecular Medicine and Biobanking, University of Malta, MSD 2080 Msida, Malta

**Keywords:** mitochondria, membranes, amyloid peptides, coenzyme Q10, lipid vesicles, aggregation, leakage

## Abstract

Mitochondria possess a double-membrane envelope which is susceptible to insult by pathogenic intracellular aggregates of amyloid-forming peptides, such as the amyloid-beta (1-42) (Aβ42) peptide and the human islet amyloid polypeptide (hIAPP). The molecular composition of membranes plays a pivotal role in regulating peptide aggregation and cytotoxicity. Therefore, we hypothesized that modifying the physicochemical properties of mitochondrial model membranes with a small molecule might act as a countermeasure against the formation of, and damage by, membrane-active amyloid peptides. To investigate this, we inserted the natural ubiquinone Coenzyme Q10 (CoQ10) in model mito-mimetic lipid vesicles, and studied how they interacted with Aβ42 and hIAPP peptide monomers and oligomers. Our results demonstrate that the membrane incorporation of CoQ10 significantly attenuated fibrillization of the peptides, whilst also making the membranes more resilient against peptide-induced permeabilization. Furthermore, these protective effects were linked with the ability of CoQ10 to enhance membrane packing in the inner acyl chain region, which increased the mechanical stability of the vesicle membranes. Based on our collective observations, we propose that mitochondrial resilience against toxic biomolecules implicit in protein misfolding disorders such as Alzheimer’s disease and type-2 diabetes, could potentially be enhanced by increasing CoQ10 levels within mitochondria.

## 1. Introduction

Protein misfolding disorders (PMDs) are diseases in which the central pathogenic event is represented by the misfolding and aggregation of soluble native peptides into amyloid fibrils with a characteristic cross-β structure, which gradually accumulate as intracellular or extracellular protein deposits [1,2]. Examples of such proteinopathies include Alzheimer’s disease (AD) and type-2 diabetes mellitus (T2DM), which are driven by the aggregation of the amyloid-beta (Aβ) peptide and the human islet amyloid polypeptide (hIAPP), respectively [3,4]. Along the aggregation cascade, from an intrinsically disordered peptide to highly-rigid fibrillar structures, aggregate assemblies sample metastable partially folded intermediates referred to as ‘oligomers’ [5]. These likely constitute the major toxic species in disease progression, in view of their membrane-disturbing properties as a result of their small size and exposed hydrophobic moieties [6,7]. For instance, Aβ and hIAPP oligomers are able to porate and destabilize cellular membranes using mechanisms reminiscent of antimicrobial peptides [8,9,10]. The interaction between amyloid aggregate complexes and lipid bilayers is twofold: while membrane-active aggregates disrupt bilayer integrity, membranes themselves can serve as catalytic platforms for the amyloid formation cascade, accelerating the processes of aberrant protein folding and fibrillar assembly [11,12]. These interactions are largely dictated by the physico-chemical properties of the bilayer, including lipid composition, charge, and curvature [13,14]. In particular, the presence of anionic phospholipids drives the initial binding event for cationic peptides such as hIAPP and Aβ, and also strongly favor aggregation on membrane surfaces [13].

Mitochondrial membranes are uniquely enriched with the anionic phospholipid cardiolipin (CL). Unlike other phospholipids, CL is a dimeric phospholipid characterized by two negatively charged phosphates in its headgroup linked by a glycerol bridge to four acyl chains. The relatively small size of the polar headgroup compared to the acyl region gives rise to a wedge-shaped structure for CL that imparts a negative curvature in membranes [15]. The inner mitochondrial membrane (IMM) and mitochondrial contact sites (formed by close apposition of the outer and inner mitochondrial membranes) are regions particularly abundant in CL (15–20%) [16]. Beyond its structural role in maintaining the morphology of cristae (highly curved folds in the IMM), CL also supports critical functions such as mitochondrial dynamics, respiratory chain supercomplex formation, and is implicated in the regulation of mitophagy and apoptosis [17]. The unique lipid composition, anionic character, and incidence of high-curvature regions possessed by mitochondrial membranes may facilitate association with amyloid peptides, especially oligomeric forms [18,19]. Mounting evidence indicates that amyloidogenic peptides like Aβ and hIAPP may accumulate within this subcellular compartment, generating mitochondrial dysfunction and ultimately triggering cell death [20,21,22,23]. Indeed, mitochondrial dysfunction is described as a central hallmark of both T2DM and neurodegenerative diseases [24,25]. Recently, Aβ oligomers were reported to increase the conductance of the voltage-dependent anion-selective channel 1 (VDAC1) found in the outer membrane of mitochondria and enhance its affinity for Ca^2+^ ions; these effects correlated with impaired mitochondrial respiration in SH-SY5Y neuroblastoma cells and an overall decrease in cell viability [26].

While PMDs continue to rise in incidence due to aging populations and lifestyle changes, at the same time, the development of effective, disease-modifying treatments for these maladies has proven to be exceptionally challenging. To date, despite the considerable efforts to develop therapies which directly target the aggregation of amyloidogenic peptides such as Aβ and hIAPP, clinical outcomes have largely been disappointing [27]. Given that biological membranes stand out as critical extrinsic factors in amyloid-induced cytotoxicity, they may be considered as compelling therapeutic targets for the treatment of PMDs. In this context, the use of small molecules to modulate the membrane’s properties might provide an innovative approach to mitigate harmful interactions between amyloid peptides and cellular membranes [28]. We hypothesize that modifying the physicochemical properties of vulnerable mitochondrial membranes, for instance through the incorporation of small-molecule compounds, could potentially modulate amyloid–membrane interactions, and in turn alleviate their associated toxicity.

As a ‘proof-of-concept’ study, here we incorporated the natural molecule Coenzyme Q10 (CoQ10) into mitochondrial-like membranes and determined its impact on the aggregation of the Aβ(1-42) (Aβ42) and human IAPP peptides, as well as on membrane-induced destabilization by oligomeric preparations of these peptides. CoQ10, also known as ubiquinone, is a key lipid-soluble component of the mitochondrial electron transport chain, shuttling electrons between complexes I and II to complex III. It is therefore most highly concentrated in the IMM [29]. In addition to CoQ10’s well-known antioxidant properties, it also plays a role in enhancing stability in membrane environments, increasing the phospholipid order [30,31,32]. To test our hypothesis, we ‘bioengineered’ model lipid vesicles harboring 15% CL, designed to mimic the phospholipid composition of the IMM and mitochondrial contact sites, with the additional incorporation of CoQ10. Thioflavin-T (ThT) assays were employed to monitor the fibrillar aggregation kinetics of the Aβ42 and hIAPP peptides in the presence of these CoQ10-incorporating vesicles. Additionally, the resilience of the CoQ10-enriched vesicles to amyloid-induced membrane damage was determined using dye leakage assays. This enabled us to correlate the behavior of the bioengineered vesicles with changes in membrane fluidity, a key factor influencing amyloid–membrane interactions.

## 2. Materials and Methods

### 2.1. Chemicals

Calcium chloride (CaCl_2_), CoQ10, dimethyl sulfoxide (DMSO), 1,6-diphenyl-1,3,5-hexatriene (DPH), 6-dodecanoyl-2-dimethylamine-naphthalene (Laurdan), ethanol, ethylenediamine tetra-acetic acid (EDTA), ionomycin, 4-morpholinepropanesulfonic acid (MOPS), potassium chloride (KCl), Thioflavin T (ThT), trifluoroacetic acid (TFA), and tris(hydroxymethyl) aminomethanine (TRIS) were purchased from Sigma-Aldrich (Munich, Germany).

### 2.2. Preparation of Aβ42 and hIAPP Oligomers

Recombinant human Aβ42 peptide (rPeptide) stock solutions were prepared by dissolving the pure peptide in 10 mM NaOH at a concentration of 200 μM. Lyophilized hIAPP(1-37) (Abcam, Cambridge, UK) was resolubilized in 100% DMSO and 0.1% TFA at 1.4 mM. Peptide samples were flash-frozen in liquid nitrogen before storing at −80 °C in LoBind epitubes (Eppendorf, Hamburg, Germany). Peptide stocks were thawed only once immediately before the experiments were started. For preparation of oligomer-rich Aβ42 peptides, 45 μM monomeric Aβ42 was aggregated for 2 h in sterile 100 mM KCl and 10 mM MOPS (pH 7.4) buffer at 37 °C, according to previously established protocols [33,34]. Predominantly oligomeric hIAPP was prepared by incubating the fresh peptide (10 μM) in a 96-well plate in KCl (100 mM) and MOPS (10 mM) buffer at pH 7.4, and aggregated in a thermo-shaker for 20 min at 37 °C with a speed of 450 rpm [35]. Oligomeric samples were used immediately, or stored at −80 °C to halt further assembly.

### 2.3. Large Unilamellar Vesicles (LUVs) Preparation

The phospholipids L-α-phosphatidylcholine (egg PC), L-α-phosphatidylethanolamine (egg PE), L-α-phosphatidylinositol (soy PI), and 15% bovine heart cardiolipin (CL) were all purchased from Avanti Polar Lipids (Alabaster, AL, USA) dissolved in chloroform. Lipid mixtures were prepared in a glass tube, using the following phospholipid combination to approximate the composition of the IMM and mitochondrial contact sites: 40PC/35PE/10PI/5PS/15CL mol/mol (15% CL) [36,37]. CoQ10 (1 mg/mL in chloroform) was also added to the lipid mixture when preparing CoQ10-incorporating liposomes (5 mol%) [31,32]. The chloroform was then removed to obtain a dry lipid film, by evaporation with nitrogen gas followed by drying under vacuum for a minimum of 2 h to completely remove any residual solvent. Next, the lipid film was resuspended by dispersion and brief vortexing in 100 mM KCl and 10 mM MOPS (pH 7.4) buffer, and the resulting homogenous milky lipid suspension left standing for 1 h to ensure full hydration. Multilamellar vesicles were subjected to ultrasonication for 10 min and subsequently passed 19 times through a mini-extruder (Avanti Polar Lipids, Alabaster, AL, USA) equipped with 0.1 um polycarbonate membranes to obtain LUVs. For leakage assays, LUVs were loaded with the Oregon Green^®^ 488 BAPTA-1 fluorophore (OG; ThermoFisher Scientific, Waltham, MA, USA) by hydrating the lipid film with liposome buffer containing 50 µM OG. Unencapsulated dye was then removed using the detergent-dialysis method [38]. Liposome samples were kept at 4 °C for a maximum of 1 week. The effective diameter and uniformity of the liposome preparation was checked before each use, using dynamic light scattering on a Zetasizer Nano S (Malvern Panalytical, Malvern, Worcestershire, UK).

### 2.4. Evaluation of Membrane Fluidity Changes in LUVs

Membrane fluidity was analyzed by fluorescence polarization (FP) measurements using Laurdan and DPH probes, which localize at different depths of the bilayer: Laurdan incorporates at the aqueous membrane surface, while DPH partitions in the inner hydrophobic core [39]. Stock solutions of 2 mM Laurdan and 2 mM DPH were prepared by dissolving in DSMO and stored in the dark. To allow for incorporation of the probes into the liposomal membranes, LUVs (100 μM) in 100 mM KCl and 10 mM MOPS (pH 7.4) buffer were incubated with 5 µM Laurdan or DPH for 30 min at 45 °C (in darkness). The liposomes (50 µM) were then pipetted into 96-well half-area, low-binding, clear-bottomed plates (Corning 3881). The FP of the dyes was registered using a TECAN Infinite^®^ 200 PRO (Salzburg, Austria) plate reader equipped with the appropriate filters (DPH: ex/em 355/430 nm; Laurdan: ex/em 360/450 nm). Before FP measurements, the plate was shaken orbitally for 30 s at an amplitude of 2.5 mm to ensure sample homogeneity. During temperature ramping experiments, the temperature was increased from 24 °C to 42 °C, in increments of 2 °C with a 10 min equilibration time following each increase to ensure temperature stability. FP measurements (mP) correlate inversely with membrane fluidity [40]. In preliminary experiments, it was confirmed that CoQ10 did not artefactually alter FP readings in the absence of LUVs.

### 2.5. Thioflavin T Kinetics Assay for Peptide Aggregation

Peptide aggregation kinetics were followed using the Thioflavin T (ThT) assay, a well-established method for the detection and quantification of amyloid fibril formation [41]. The assay was started by adding 10 μL of Aβ42 or hIAPP (10 μM peptide) to 190 μL of a solution containing 20 μM ThT, LUVs (10 μM or 50 μM lipids), 100 mM KCl and 10 mM MOPS/Tris buffer (pH 7.4). For the preparation of 15% CL LUVs pre-incubated with CoQ10, CoQ10 (1 mM, dissolved in 100% DMSO) was added to 50 μM 15% CL LUVs at a final concentration of 2.5 μM and incubated for 20 min at RT. This concentration was selected to match the estimated amount of CoQ10 incorporated in the CoQ10-incorporating liposomes. Samples were prepared in a 96-well black, clear-bottom microplate (Corning no. 3631) which was sealed and incubated at 37 °C in a Thermomixer Comfort ^®^ (Eppendorf, Hamburg, Germany) under 450 rpm shaking conditions. At regular intervals, the ThT fluorescence intensity was recorded in a BioTek FLx800 plate reader (λex = 450 nm and λem = 485 nm). The assays were performed in triplicate using different peptide stock solutions.

### 2.6. Liposome Membrane Permeability and Osmotic Stress Assays

Liposome leakage experiments were conducted on OG-encapsulated lipid vesicles, in standard 96-well black flat-bottom plates (Corning no. 3915). The peptides at 5 μM were added to a mixture of 25 μM OG-loaded LUVs in buffer (100 mM KCl, 10 mM MOPS/Tris, 1 mM EDTA (pH 7.4)) containing 0.1 mM CaCl_2_. For the osmotic stress experiments, 0.4 M KCl was used in the buffer. An increase in fluorescence resulting from OG-calcium binding indicated loss of membrane integrity [33]. Fluorescence was measured every 5 min for 30 min using excitation and emission filters of 485 nm and 528 nm, respectively. The temperature was set at either room temperature (24 °C) or physiological temperature (37 °C). The percent leakage of fluorescent OG dye from lipid vesicles was normalized according to the following equation:

Permeabilization (% of ionomycin) = (*F_t_* − *F*_0_)/(*F_max_* − *F*_0_) × 100

where *F_t_* is the measured fluorescence intensity at time *t*, *F*_0_ denotes the fluorescence of intact liposomes before the addition of peptide or 0.4 M KCl, *F_max_* represents the maximum fluorescence intensity obtained with the addition of the calcium ionophore ionomycin (10 μM) (total vesicle disruption). Readings were always taken in triplicate.

### 2.7. Statistical Analysis

Statistical comparisons between mean values were performed using the unpaired two-tailed Student’s *t*-test for pairwise comparisons, and one-way or two-way ANOVA tests followed by Bonferroni’s post hoc correction for multiple comparisons. Normality was assessed using the D’Agostino and Pearson tests. For non-parametric data with multiple comparisons, the Kruskal–Wallis test was applied. Data are presented as the mean ± standard error of the mean (SEM), with the number of replicate experiments specified in each figure legend. The statistical significance threshold was defined as * *p* < 0.05, ** *p* < 0.01, *** *p* < 0.001, **** *p* < 0.0001. All statistical analyses were conducted using GraphPad Prism 10 software package (GraphPad Software, La Jolla, CA, USA).

## 3. Results

### 3.1. CoQ10 Membrane Incorporation Enhances Packing Order in CL-Containing Liposomes

**Figure 1:**
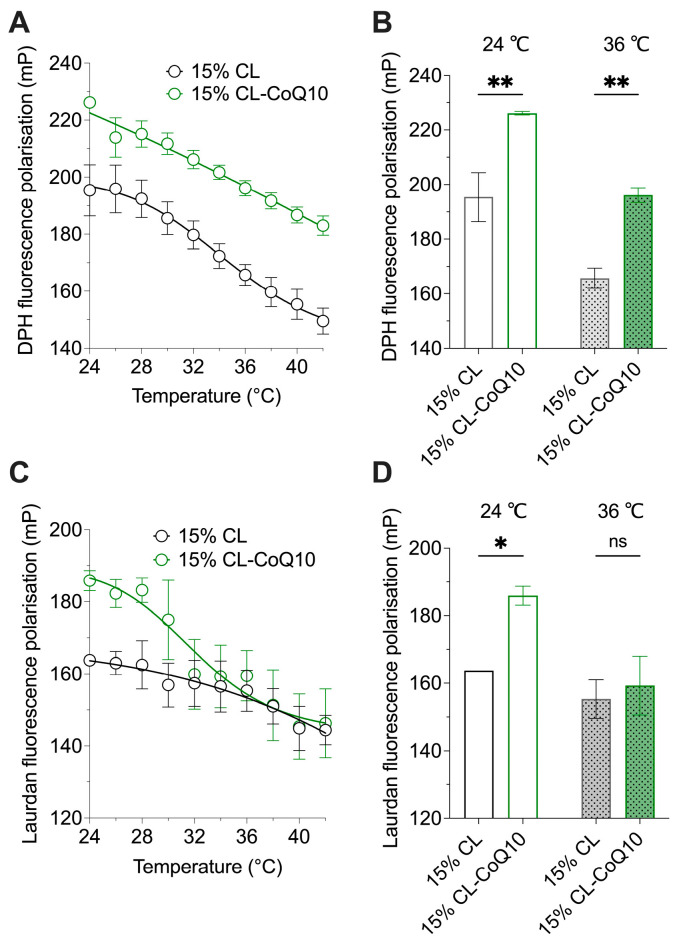
Effect of CoQ10 incorporation on membrane fluidity of 15% CL liposomes. Changes in membrane packing were analyzed using fluorescent probes DPH (for inner acyl chain region) and Laurdan (for outer water–lipid interface region). The polarization of (**A**) DPH and (**C**) Laurdan fluorophores in the membranes of 15% CL and 15% CL-CoQ10 LUVs was measured from 24 °C to 42 °C (*n* = 3). Data were extracted from these graphs to create bar charts comparing (**B**) DPH and (**D**) Laurdan mP values at 24 °C and 36 °C (*n* = 3–5). Values represent means ± SEM; two-way ANOVA with Bonferroni’s multiple comparisons test (ns, *p* > 0.05, * *p* < 0.05, ** *p* < 0.01).

CoQ10 is a lipophilic isoprenoid residing mainly within the cell in the inner membrane of mitochondria, typically at 0.5–2 mol% relative to the lipid content of the membrane [42]. To allow a clear assessment on how the integration of CoQ10 into the liposome membrane would influence membrane structure and behaviour, mito-mimetic lipid vesicles were synthesized incorporating 5 mol% CoQ10 (15% CL-CoQ liposomes). FP experiments employing Laurdan and DPH probes were performed to monitor bilayer fluidity of the 15% CL-CoQ vesicles at the lipid headgroup region and within the membrane’s hydrophobic core, respectively. The effect of CoQ10 incorporation on membrane fluidity was tracked across a temperature gradient, since increasing temperatures promote more disordered—and hence more fluid—membranes [43]. Overall, incorporation of CoQ10 in 15% CL membranes resulted in a significant restriction in the mobility of the phospholipid acyl chains, as indicated by higher DPH FP values compared to pure 15% CL liposomes across the whole tested temperature range (24–42 °C) (Figure 1A). Specifically, the extracted DPH data at 24 °C and 36 °C showed significantly increased DPH FP values (by ~15%) with respect to the 15% CL-CoQ10 LUVs (*p* = 0.0031 and *p* = 0.0053, respectively) (Figure 1B). Probing the outer headgroup region with Laurdan dye, the 15% CL-CoQ10 LUVs also displayed increased rigidity in this region of the bilayer (*p* = 0.0119) (Figure 1C), however, only at temperatures up to 30 °C; at higher temperatures, mP levels were not different from pure 15% CL liposomes (Figure 1D). Together, the FP results show that while CoQ10 inclusion in the membrane robustly maintained acyl chain order across all temperatures, the rigidifying effect was lost within the outer bilayer at physiologically relevant (~37 °C) temperatures. In summary, because Laurdan and DPH probes partition into different regions of the bilayer (outer and inner, respectively), we may conclude from the fluidity experiments that CoQ10 maintains membrane stability at higher temperatures in the inner, but not outer, regions of the bilater. We correlate this effect with the highly hydrophobic nature of CoQ10, in which the lipophilic isoprenoid tail interacts strongly with the fatty acyl chains of the membrane phospholipids—rather than their polar headgroups—hence driving its localization to the inner region of the membrane [44].

Next, we wanted to determine the observed impact on membrane fluidity of CoQ10 incorporation compared with CoQ10 incubation, i.e., wherein 15% CL liposomes were exposed to CoQ10 in buffer. In the latter scenario, CoQ10 (even up to 50 μM) exerted no impact on membrane fluidity at all (Figure S1). Altogether, these results stress the necessity of integrating CoQ10 in the lipid bilayer for a successful induction of a more ordered membrane environment.

### 3.2. CoQ10-Incorporating LUVs Suppress Amyloid Peptide Aggregation

Lipid membranes, and especially those with an overall anionic surface charge, are known to favor the aggregation pathways of both Aβ42 and hIAPP peptides [45,46,47,48]. We therefore wanted to investigate whether the rigidified membrane structure of mito-mimetic membranes supplemented with CoQ10 observed previously could influence membrane-catalyzed amyloid peptide aggregation. With this aim, the aggregation of Aβ42 and hIAPP peptides into a cross-β-sheet fibrillar structure was followed by ThT fluorescence in the presence of 15% CL and 15% CL-CoQ LUVs. ThT is commonly used to monitor in vitro fibril formation by amyloidogenic peptides and proteins since it binds to accessible β-strands along the long axis of the fibrils [49]. Apart from comparing ThT fibril formation kinetics in the presence of 15% CL and 15% CL-CoQ LUVs, in these experiments we also included 15% CL LUVs that had been pre-incubated with CoQ10. This would allow us to determine the effect of CoQ10 when present in the extra-vesicular solution, in comparison to its effect when incorporated in the 15% CL-CoQ10 LUVs.

**Figure 2:**
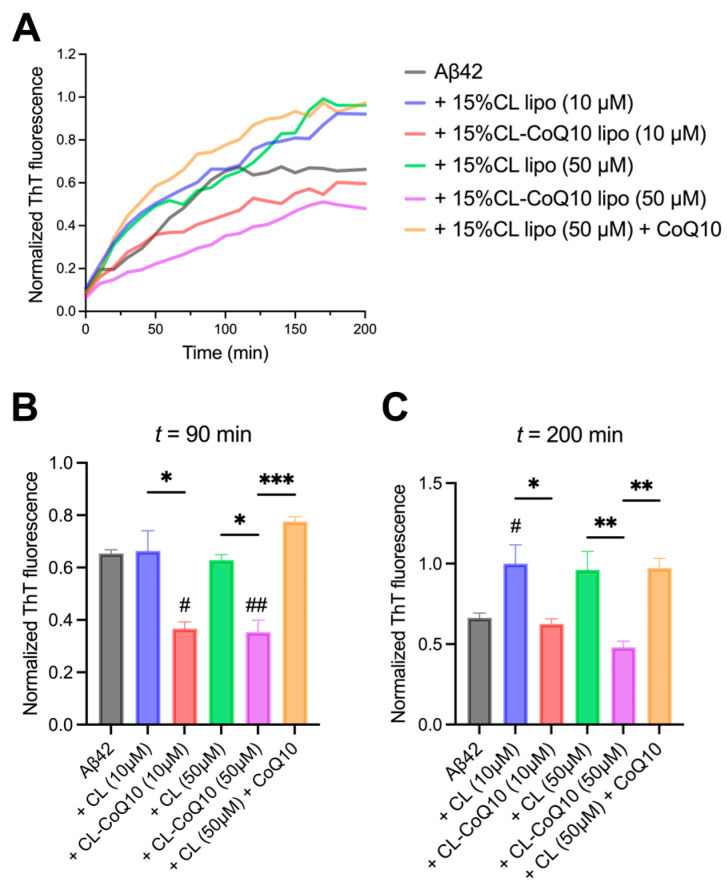
Aβ_42_ fibrillization in the presence of 15% CL and 15% CL-CoQ10 LUVs. (**A**) ThT kinetic fluorescence curves of 10 μM Aβ_42_ aggregated alone, and with 15% CL, 15% CL-CoQ10 LUVs (10–50 μM), as described in Section 2. Also included were 15% CL LUVs (50 μM) pre-incubated for 20 min with 5 μM CoQ10. Mean ThT values in solid lines. (**B**,**C**) ThT fluorescence data points were extracted from the kinetic graphs at mid-log (90 min) and endpoint (200 min) readings. Values reflect means ± SEM (*n* = 4–6); one-way ANOVA with Bonferroni’s multiple comparisons test between marked pairs (* *p* < 0.05, ** *p* < 0.01, *** *p* < 0.001); or between Aβ42 and the indicated sample (# *p* < 0.05, ## *p* < 0.01).

Monomeric Aβ42 was incubated in the presence of 1:1 and 5:1 lipid–protein ratios (L:P), corresponding to 10 μM and 50 μM LUVs (for both 15% CL and 15% CL-CoQ10). The kinetics of Aβ42 assembly into fibrils were strongly inhibited by 15% CL-CoQ10 liposomes compared to their respective control 15% CL vesicles, with a more robust inhibitory effect at the higher L:P (5:1) ratio (Figure 2A). ThT fluorescence intensity data extracted at around mid-log (90 min) of the aggregation curves revealed already a reduction of ~55% in β-sheet-rich Aβ42 aggregate formation in the presence of CoQ10-supplemented LUVs when compared to Aβ42 aggregation in the presence of control liposomes (10 μM, *p* = 0.0105; 50 μM, *p* = 0.0027) (Figure 2B). Aβ42 fibril formation with 15% CL-CoQ10 liposomes was slowed down even in comparison to aggregation of the peptide alone, i.e., in the absence of any vesicles (10 μM, *p* = 0.0131; 50 μM, *p* = 0.0005). This strong inhibition by 15% CL-CoQ10 LUVs was maintained to the endpoint of the aggregation process at 200 min (Figure 2C). Moreover, the aggregation profile of the Aβ42 peptide in the presence of CoQ10-incorporating liposomes differed significantly from that observed with CoQ10-incubated 15% CL vesicles. In the latter case, pre-incubation of 15% CL LUVs for 20 min with CoQ10 failed to have any effect on the suppression of Aβ42 fibrillization (Figure 2B,C). Thus, we may infer that modulation of Aβ42 peptide aggregation by CoQ10 necessitates its presence directly in the mito-mimetic membrane, which correlates with the rigidification of the bilayer.

**Figure 3:**
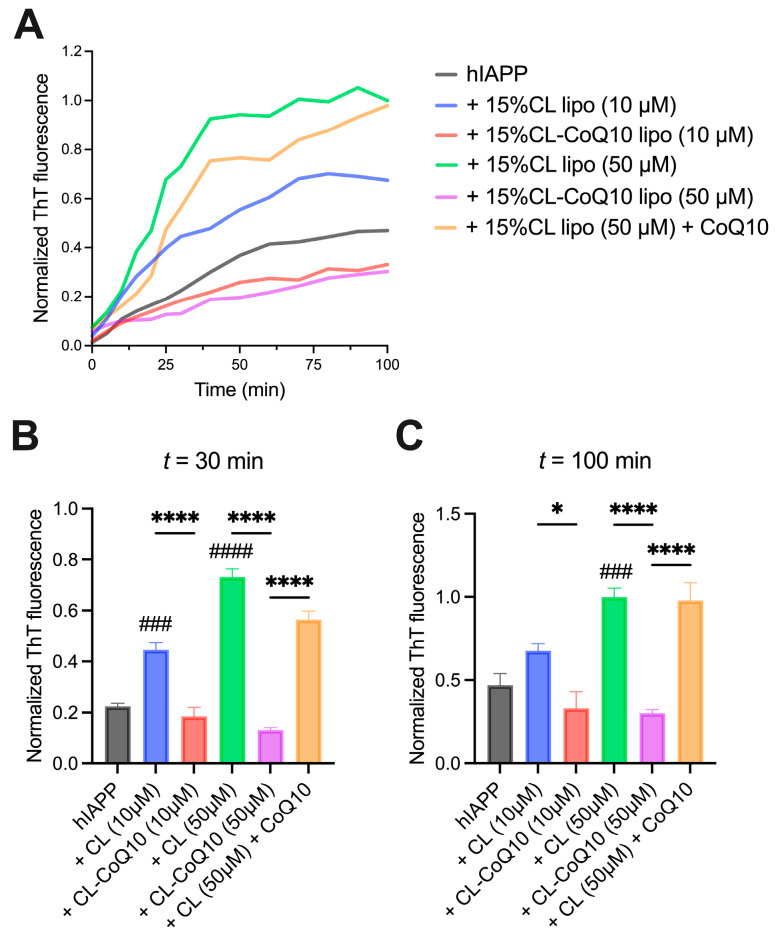
Human IAPP fibrillization in the presence of 15% CL and 15% CL-CoQ10 LUVs. (**A**) ThT kinetic fluorescence curves of 10 μM hIAPP aggregated alone, and with 15% CL, 15% CL-CoQ10 LUVs (10–50 μM), as described in Section 2. Also included were 15% CL LUVs (50 μM) pre-incubated for 20 min with 5 μM CoQ10. Mean ThT values in solid lines. (**B**,**C**) ThT fluorescence data points were extracted from the kinetic graphs at mid-log (30 min) and endpoint (100 min) readings. Values reflect means ± SEM (*n* = 4); one-way ANOVA with Bonferroni’s multiple comparisons test between marked pairs (* *p* < 0.05, **** *p* < 0.0001); or between hIAPP and the indicated sample (### *p* < 0.001, #### *p* < 0.0001).

We next repeated the above set of experiments with hIAPP to assess the impact of the CoQ10-incorporating liposomes on the fibrillization kinetics of this peptide. Hence, parallel to the previous experimental approach, hIAPP fibrillization was monitored with ThT in the presence of 15% CL and 15% CL-CoQ LUVs at 1:1 and 5:1 L:P ratios. Firstly, the presence of CL-enriched LUVs significantly accelerated and promoted hIAPP fibril formation, in comparison to hIAPP alone (Figure 3A). This pro-aggregating effect was particularly pronounced at the higher L:P ratio (50 μM 15% CL-CoQ10 LUVs) and resulted in an ~2-fold increase in ThT-positive fibrils at the endpoint of the aggregation (*p* = 0.0002) (Figure 3C). Remarkably, however, despite this powerful pro-aggregative influence of the 15% CL liposomes, hIAPP β-sheet assembly was strongly suppressed by the 15% CL-CoQ10 liposomes throughout the fibrillization process (Figure 3B). At endpoint, there was a 50% and 70% decrease in hIAPP fibril formation with 10 μM (*p* = 0.0335) and 50 μM (*p* < 0.0001) 15% CL-CoQ10 LUVs, respectively (Figure 3C). On the other hand, as previously observed with Aβ42, CoQ10-incubated 15% CL vesicles did not inhibit hIAPP aggregation; in this case, both the mid-log and endpoint ThT values were similar to those of the 15% CL vesicles alone (Figure 3B,C). Altogether, these observations suggest that CoQ10 protects against amyloidogenic peptide aggregation and this is dependent on its integration into the liposomal membrane.

### 3.3. CoQ10-Incorporating LUVs Display Enhanced Resilience to Peptide-Induced Vesicle Permeabilization and Osmotic Stress

**Figure 4:**
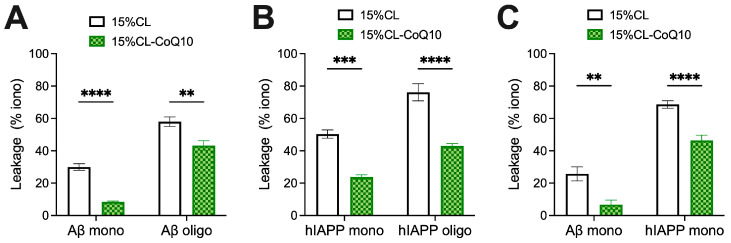
Permeabilization of LUVs by Aβ42 and hIAPP species. (**A**,**B**) 25 μM OG-filled LUVs (15% CL and 15% CL-CoQ10) were incubated at room temperature (24 °C) with either fresh (monomeric) or aggregated (oligomeric) 5 μM Aβ42 and hIAPP species, respectively, as described in Section 2. Leakage was quantified after 30 min incubation, calculated as a percentage (%) of the maximal ionomycin-induced flourescence. (**C**) Leakage assays using Aβ42 and hIAPP monomers were repeated at physiological temperature (37 °C). Values represent means ± SEM (*n* = 4–8); two-way ANOVA with Bonferroni’s multiple comparisons test (** *p* < 0.01, *** *p* < 0.001, **** *p* < 0.0001).

Mito-mimetic vesicles, possessing CL-rich membranes, have been reported to be more susceptible to disruption by amyloidogenic proteins than those lacking CL [38,50]. This is likely to be related to an increased membrane fluidity and greater occurrence of membrane defects associated with higher concentrations of CL [51,52]. Therefore, we next sought to determine whether the supplementation of our mito-mimetic vesicle membranes with CoQ10 would, apart from inhibiting peptide aggregation, also curtail the permeabilization efficiency of membrane-active peptides of Aβ42 and hIAPP. In our experimental design, 15% CL and 15% CL-CoQ10 vesicles were loaded with OG dye and exposed to either fresh or aggregated (oligomer-rich) samples of 5μM Aβ42 and hIAPP. Oligomers were included in view of the fact that they are considered as the most highly membrane-active aggregate species, underpinning their cytotoxicity [53]. Indeed, exposure of 15% CL liposomes to oligomeric Aβ42 induced almost double the vesicle permeabilization compared to monomeric Aβ42 (Aβ42 mono: 30.6 ± 6.0%; Aβ42 oligo: 58.0 ± 7.8%), thus confirming the high membrano-toxicty of the oligomeric form (Figure 4A). Notably, however, incorporation of CoQ10 significantly reduced the dye leakage induced by both Aβ42 monomers (by 21.6%; *p* < 0.001) and Aβ42 oligomers (by 14.8%; *p* = 0.0041) (Figure 4A). Hence, CoQ10 incorporation effectively preserved bilayer integrity against both fresh and oligomeric Aβ42, mitigating membrane disruption.

In a similar set of experiments, OG-loaded LUVs were exposed to fresh and oligomeric hIAPP. Again, exposure to oligomeric hIAPP triggered significantly higher levels of dye leakage than the monomeric sample. Specifically, the addition of fresh hIAPP induced 50.4 ± 8.7% dye leakage, while the oligomeric sample resulted in 76.1 ± 15.8% leakage from 15% CL LUVs (Figure 4B). Consistent with the Aβ leakage data, the ‘bioengineered’ liposomes incorporating CoQ10 successfully mitigated dye leakage in the presence of both hIAPP species, compared to the control vesicles. Upon exposure to fresh hIAPP, CoQ10-incorporating LUVs exhibited approximately twofold less permeabilization (*p* = 0.0004; Figure 4B). In the presence of pre-aggregated hIAPP oligomers, leakage of OG from the bioengineered liposomes decreased by 18%, compared to the control 15% CL population (*p* < 0.0001; Figure 4B). Together, the results suggest that the presence of CoQ in mito-mimetic vesicles effectively counters bilayer disruption upon exposure to fresh and oligomeric peptide samples.

We next asked the question as to whether a higher temperature might compromise the protection afforded by CoQ10 in the membrane. This, in view of the fact that the DPH/Laurdan experiments had shown that higher temperatures lead to increased membrane fluidity (Figure 1)—and hence, potentially, a greater degree of permeabilization by the peptides. Considering Aβ42 monomers, permeabilization of both the 15% CL (25.8 ± 8.7%) and 15% CL-CoQ10 (6.7 ± 5.2%) liposomes at 37 °C did not differ appreciably from room temperature (24 °C) conditions (Figure 4C). Hence, the CoQ10-supplemented mito-mimetic vesicles maintained their protection at the higher physiological temperature (15% CL vs. 15% CL-CoQ10, *p* = 0.0026).

On the other hand, with hIAPP monomers, increased dye leakage was observed at 37 °C from both 15% CL (68.6 ± 6.1%) and 15% CL-CoQ10 (46.4 ± 7.4%) LUVs when compared to data obtained at 24 °C. Nevertheless, the enhanced resilience of CoQ10-supplemented membranes was still extremely significant at this higher temperature (15% CL vs. 15% CL-CoQ10, *p* < 0.0001). Notably, the packing density in the acyl region of 15% CL liposomes was increased by the insertion of CoQ10, in the temperature-ramping experiments (Figure 1). Considering this, one may infer a correlation between (increased) acyl bilayer order in the hydrophobic core and (decreased) membrane vulnerability to disruption by amyloid aggregates—the former being modulated by the presence of CoQ10 in the bilayer.

**Figure 5:**
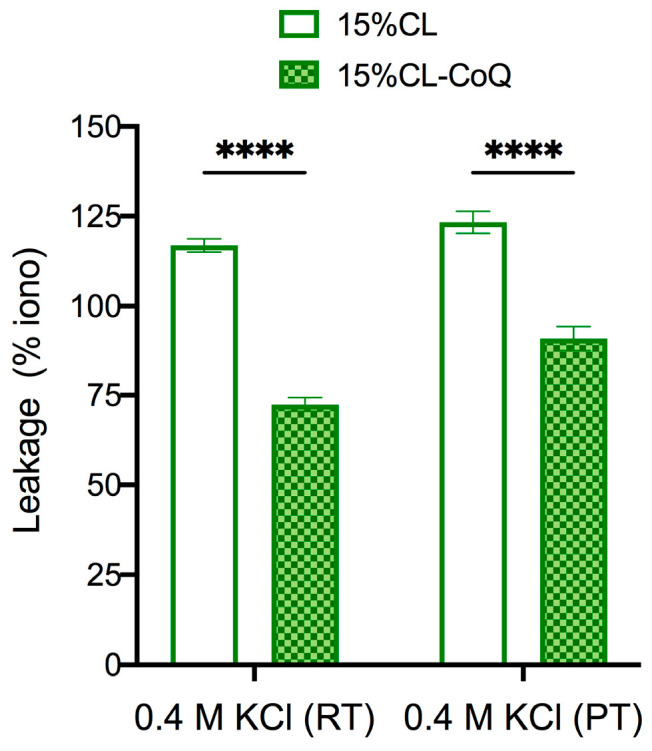
Permeabilization of LUVs by osmotic stress. 15% CL and 15% CL-CoQ10 liposomes loaded with OG were incubated with 0.4 M KCl at room temperature (RT; 24 °C) and physiological temperature (PT; 37 °C). The maximal leakage over 60 min was calculated as a percentage of ionomycin. Values reflect means ± SEM (*n* = 3); two-way ANOVA with Bonferroni’s multiple comparisons test (**** *p* < 0.0001).

To reinforce the hypothesis that CoQ10 strengthens the membrane of mito-mimetic vesicles, 15% CL and 15% CL-CoQ10 liposomes were subjected to osmotic stress using buffer solutions containing 0.4 M KCl. It would be expected that an increased stability of the vesicle membrane would translate into less dye leakage upon osmotic stress. Experiments were conducted at room (24 °C) and physiological (37 °C) temperatures. In support of this notion, the bioengineered CoQ10 liposomes demonstrated greater membrane stability compared to the native 15% CL liposomes when subjected to 0.4 M KCl stress, at both 24 °C (leakage decreased by 45%; *p* < 0.0001) and 37 °C (leakage decreased by 32%; *p* < 0.0001) (Figure 5). Hence, the data support the conclusion that CoQ10 provides mechanical stability to the mito-mimetic membranes.

## 4. Discussion

The physico-chemical properties of phospholipid bilayers are pivotal in governing peptide–membrane interactions, which ultimately modulate the extent of toxicity exerted by membrane-active aggregates of the Aβ and hIAPP peptides [13,14]. Alterations in lipid membrane composition, driven by aging and disease, have also been implicated in increasing membrane susceptibility to amyloid damage [54]. Mitochondrial membranes, uniquely enriched with CL, may be particularly susceptible to peptide-induced damage, for instance, by the formation of ion-conducting transmembrane pores or larger openings [19,55,56,57,58,59,60]. The fact that mitochondrial integrity is indispensable for cellular survival and function underscores the significance of exploring strategies that can protect mitochondrial membranes from amyloid toxicity. The incorporation of small-molecule compounds into mitochondrial bilayers may provide a relatively unexplored approach to modulate aberrant peptide–membrane interactions [28]. In this study, we demonstrate that the incorporation of the natural ubiquinone CoQ10 into CL-rich mitochondrial model membranes stabilizes the bilayer to interfere with amyloidogenic peptide self-assembly and attenuate peptide-induced membrane disruption.

For the current investigation, mitochondrial membranes were modeled in vitro using LUVs made with defined lipid compositions that mimic the IMM and mitochondrial contact sites, having 15% CL content. This approach allowed for the use of physiologically relevant membrane systems, compared to the more usual one- or two-component simple model membranes, hence providing better translational insight. Furthermore, to allow for efficient incorporation of CoQ10 into the model membrane, the small molecule was added ab initio to the 15% CL lipid mixture. This ensured that the synthesized LUVs incorporate the isoprenoid directly solubilized within the lipid leaflet. Moreover, the absence of CoQ10 from the extra-vesicular environment allowed us to assess directly the role of CoQ10 in modulating membrane structure and behavior. A similar method was previously used to investigate the osmotic-stress tolerance of artificial liposomes containing ubiquinone-8 [31].

The existing literature describes that the inclusion of CL in bilayers, besides inducing negative curvature, increases bilayer fluidity and deformability [61,62]. Interestingly, in our experiments, the incorporation of CoQ10 had a striking effect on membrane fluidity. This is evidenced by the FP investigations using DPH and Laurdan probes, which revealed a rigidified acyl chain environment in the 15% CL-CoQ10 vesicles across a broad temperature range. On the other hand, mere co-incubation of CoQ10 with pre-formed ‘native’ 15% CL vesicles did not affect membrane fluidity, implying a necessity for CoQ10 to reside within the hydrophobic core of the bilayer for a chain-ordering effect. These findings align with previous observations which report similar chain-ordering and membrane-condensing effects of CoQ10 [30,32].

More importantly, the increased acyl chain packing of 15% CL-CoQ10 LUVs correlated with a significant inhibitory effect on both Aβ and IAPP peptide aggregation. This outcome suggests that the inclusion of CoQ10 in mitochondrial-like membranes powerfully impeded CL-induced aggregation by the bilayer. Such an effect is particularly significant when one considers that the ‘native’ IMM-like liposomes (i.e., without incorporated CoQ10) exhibited a notable catalytic effect on peptide aggregation, as evidenced by the reduced lag times and/or increased ThT fluorescence maxima when compared to aggregation of the peptide alone in solution. This pro-aggregative effect is consistent with the established role of anionic membranes, such as those containing CL, in promoting peptide misfolding and assembly [45,63,64]. Additionally, CL-induced membrane curvature and packing defects may have profound implications for direct lipid–peptide interactions. For instance, hIAPP has been shown to sense membrane ‘wedging’ and localize to mitochondrial cristae [65]. Localization to mitochondrial cristae has also been demonstrated for the Aβ42 peptide, both in vitro and in vivo [20,66]. Apart from these two amyloidogenic peptides, it has recently been shown that a familial mutant of the alpha-synuclein protein (A53T), the misfolding and aggregation of which is linked to Parkinson’s disease, seeds are intracellularly preferentially on mitochondrial membrane surfaces, with CL being sequestered within the aggregating lipid–protein complexes [67]. In light of the above, the rationale for using CoQ10 as a membrane-rigidifying compound to interfere with amyloid protein aggregation at mitochondrial ‘hotspots’ is indeed compelling.

The modulation of peptide–membrane interactions by CoQ10 incorporation extended to reducing the vesicle membrane’s susceptibility to peptide-induced disruption. This protective effect against vesicle permeabilization was evident against both monomeric and the more membrane-damaging oligomeric species of both peptides. Moreover, even at a higher physiological temperature (37 °C), the resilience of the ‘bioengineered’ CoQ10 liposomes still held; hence, specifically correlating with the more ordered inner acyl chain region shown by the DPH experiments. These collective observations reinforce our hypothesis that enhancing membrane packing protects the mitochondrial membrane from perturbation by amyloid peptides. A more direct link between membrane fluidity/packing and resistance to membrane damage emerged when the CoQ10-incorporating vesicles were shown to be more able to withstand rupture due to osmotic stress, implying a higher degree of mechanical membrane stabilization. Interestingly, the osmoprotective effect of CoQ10 on mito-mimetic model membranes is reminiscent of the increased osmotic-stress tolerance afforded by ubiquinone-8 (Q8) accumulation in *Eschericia coli* bacteria [31]. Indeed, CoQ10 has been described to function as “cellular armor”, providing cytoprotection of endothelial cells by delaying the insertion of Aβ_25-35_ into the plasma membrane and mitochondria [68,69].

The influence of CoQ10 on the permeability and mechanical properties of phospholipid membranes may be compared to those of cholesterol [32]. An increase in cholesterol in membranes inhibits Aβ channel insertion in liposomal and cellular membranes, whilst on the other hand, cholesterol depletion facilitates peptide insertion in membranes and pore formation [70]. Similarly, in molecular dynamics simulations, cholesterol induced increased ordering of lipids which correlated with a decrease in the insertion depth of IAPP and a decreased aggregation propensity of the peptide [71]—analogous to our observations using CoQ10 in the mito-mimetic membranes. By investigating different sterols, Zhang et al. [72] established that sterols which led to a more tightly packed membrane environment resulted in lower vesicle-binding of IAPP, decreased membrane leakage, and slowed peptide assembly. Another study investigated the effects of olesoxime, a cholesterol-like synthetic compound, on mitochondrial membrane fluidity in Huntington’s disease (HD) models [73]. Mitochondrial membranes isolated from brains of transgenic HD rat models showed a significant increase in membrane fluidity, which was restored to normal when striatal cells were treated with olesoxime, or when the transgenic rats were fed olesoxime for 12 months. Remarkably, olesoxime specifically impacted mitochondrial fluidity, while leaving plasma membranes unaffected [73]. This study therefore demonstrates the potential of pharmacologically modifying mitochondrial membranes in vivo to treat mitochondrial dysfunction in neurodegenerative disorders.

**Figure 6:**
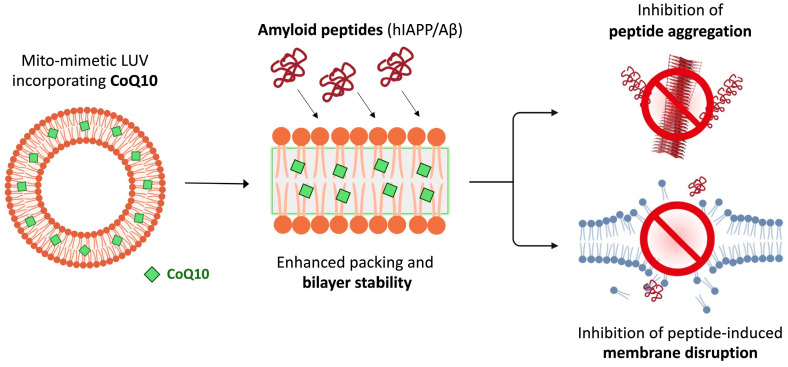
Schematic summarizing the mechanism of action of CoQ10. Incorporation of CoQ10 into mito-mimetic vesicles (15% cardiolipin content) creates a more highly ordered acyl chain environment, which contributes to (i) inhibiting fibrillar aggregation of disordered peptides like hIAPP and amyloid-β, and (ii) increased resilience of the vesicles against disruption by oligomeric species of the peptides.

Altogether, we have found that incorporation of CoQ10 into mito-mimetic vesicles: (i) enhances membrane packing across a broad temperature range, especially within the hydrophobic core of the bilayer; (ii) inhibits fibrillization of both Aβ42 and hIAPP peptides; and (iii) increases resilience of the membrane to damage caused by toxic species of the peptides. Furthermore, we correlate these effects, which are dependent on CoQ10 being embedded within the bilayer, with an increased mechanical stability of the membrane (Figure 6).

Our work thus extends the role of ubiquinones as membrane stabilizers to mitochondrial membranes, which is significant because of the central role of mitochondrial dysfunction in these diseases. However, it needs also to be acknowledged that changing the physico-chemical composition of membranes may impact a plethora of properties and functions, particularly in mitochondria where IMM structure and packing density influence mitochondrial performance [74]. In this regard, further studies are necessary in higher-order models to better understand the implications of enhancing CoQ10 content in mitochondrial membranes, both in vitro and in vivo. Notwithstanding these caveats, our work offers valuable insights that can inform the development of novel therapeutic strategies based on influencing the molecular composition of mitochondrial membranes in order to regulate peptide aggregation and cytotoxicity in amyloid-related diseases.

## Data Availability

The raw data supporting the conclusions of this article will be made available by the authors on request.

## Supplementary Materials

The following supporting information can be downloaded at: https://www.mdpi.com/article/10.3390/membranes15050148/s1, Figure S1. Effect of CoQ10 co-incubation with 15%CL liposomes on membrane fluidity.
