# Supplementary material for: Coenzyme Q10 Enhances Resilience of Mitochondrial-like Membranes Against Amyloidogenic Peptides
Source: Membranes (Basel). 2025 May 13;15(5):148. doi: 10.3390/membranes15050148 (PMC12113080; doi:10.3390/membranes15050148)
Supplement: Supplementary file 1 [file membranes-15-00148-s001.zip › membranes-3622975-supplementary.pdf]

SUPPLEMENTARY FIGURES

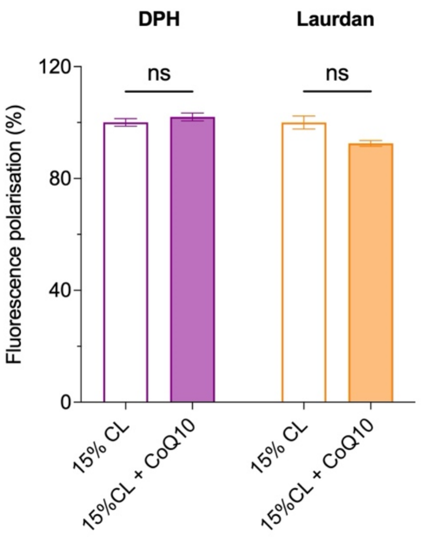

Figure S1: Effect of CoQ10 co-incubation with 15%CL liposomes on membrane fluidity. 15%CL liposomes were incubated with CoQ10 (50 μM) for 40 min at RT before measurements were taken (*n* = 3). Values represent means ± SEM; two-way ANOVA with Bonferroni's multiple comparisons test (ns, $p > 0.05$).
